# Analysis of nutritional status and influencing factors in patients with thoracoabdominal aortic dissection receiving 3D printing-assisted stent graft fenestration

**DOI:** 10.1186/s13019-023-02185-6

**Published:** 2023-03-21

**Authors:** Yan Zhou, Ying Xu, Ying Cai

**Affiliations:** Department of Vascular Surgery, Nanjing Drum Tower Hospital, The Affiliated Hospital of Nanjing University Medical School, Nanjing, 210008 China

**Keywords:** Aortic dissection, 3D printing, Stent graft fenestration, Nutritional status, Influencing factors

## Abstract

**Background:**

To investigate the nutritional status of patients with aortic dissection (AD) treated with using 3D printing-assisted stent graft fenestration and explore the important factors affecting the nutrition status of patients with different numbers of fenestrations (holes).

**Methods:**

Ninety-nine hospitalized patients with AD in a grade A tertiary hospital in Nanjing from January 2020 to December 2020 were selected as the study subjects. According to the different number of fenestrations, the patients were divided into four groups: one fenestration (group A), two fenestrations (group B), three fenestrations (group C) and four fenestrations (group D); and the nutrition status of patients in the four groups was analyzed. Then, according to whether the calories provided via infusion reached the 80% goal calories (25 kcal/kg/day) on postoperative day 5, the patients were assigned to the Reached group and Not reached group, and their inflammatory parameters, including white blood cell (WBC) and C-reactive protein (CRP), on postoperative days 1 and 5 were analyzed.

**Results:**

Compared with patients in group B (18.8%), C (19.4%) and D (6.7%), patients in group A (48.6%) had the highest rate of reaching the nutrition requirement (80% goal calories). Further, in the Reached group, WBC count and CRP concentration were significantly reduced on postoperative day 5 compared with postoperative day 1, and the proportion of patients with abnormal WBC count was significantly decreased. In contrast, although the CRP concentration on postoperative day 5 in the Not reached group was significantly lower than that on postoperative day 1, no significant changes in WBC count were observed.

**Conclusion:**

In 3D printing-assisted stent graft fenestration for AD, multiple fenestrations (holes) were associated with a low rate of reaching nutrition requirements, which might be related to imflammation. Therefore, effective nutritional support should be given to patients with multiple fenestrations after operation to improve their nutritional status and prognosis.

## Background

Aortic dissection (AD) is a pathological change that begins with aortic intima rupture, enabling blood to rush through the tear in the inner layer and move along the long axis of the aorta, separating the intima from the media, causing a septum between the true and false lumens of the aorta [1]. Although AD is a rare disease with an incidence of only one in 200,000, aortic rupture has a mortality rate as high as 34.5% [2, 3]. 3D printing-assisted stent graft fenestration are an important approach for aortic lesions involving visceral, subclavian, or carotid arteries. Contrast-enhanced cardiac CT scans from patients were post-processed and obtained the precise spatial data, then transformed and reconstructed into 3D models, finally thoracic aorta models of aortic aneurysm and aortic dissection were precisely printed by 3D printing technology [4]. It enables the anatomical structure and positional relationship between each branch artery and aneurysm visible, increasing the landing zone, and achieving optimized endovascular repair, which is an effective means of treating complex aortic diseases [5, 6].

However, surgery significantly affects the whole body, causing disruptions in the normal nutritional and immune status, which increase the risk of postoperative inflammatory response and infection in patients. Also, hypercatabolic syndrome occurring at the early stage of aortic disease may disbalance the nutritional status of patients, affecting their prognosis [7, 8]. Therefore, proper postoperative nutritional support is necessary, which not only can improve the negative nitrogen balance caused by protein metabolism disorders but also promote the recovery of immune function, improve the inflammatory response, and reduce the incidence of infection [9].

Briefly, nutritional support contributes to improving postoperative recovery and prognosis of patients. Presently, accurate nutritional support therapy is mostly used for patients in ICU. Lack of nutritional evaluation and treatment of complex aortic diseases affects patient outcomes. Therefore, this study investigated the nutritional status of patients after aortic 3D printing-assisted stent graft fenestration and explored the important factors affecting the nutritional status of patients after surgery to provide a basis for more accurate nutritional support therapy and nursing intervention in clinical practice.

## Methods

### Study subjects

**Fig. 1:**
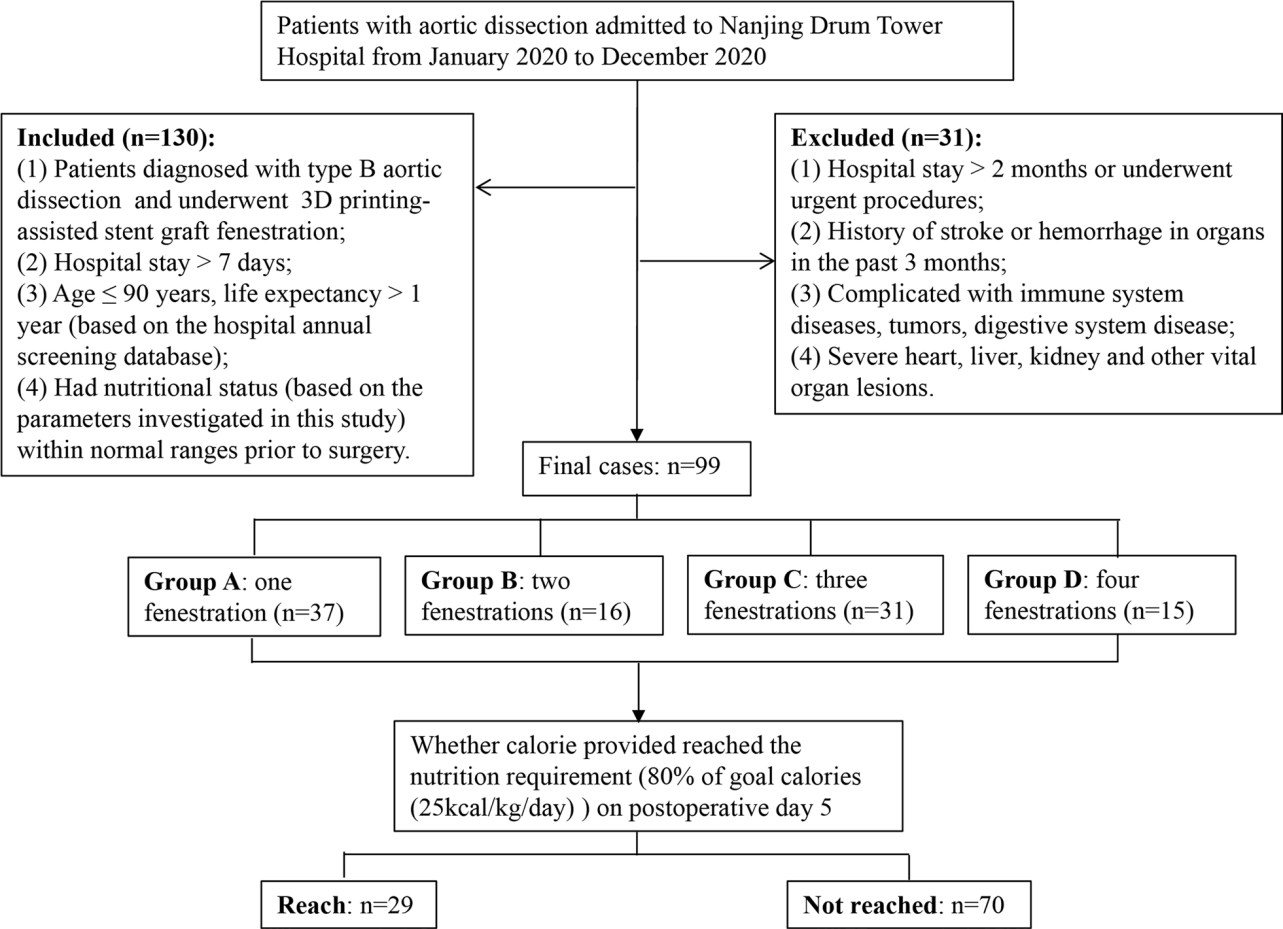
Study flow chart of the patient selection process

We initially screened the records of 130 patients with AD who were hospitalized in Nanjing Drum Tower Hospital from January 2020 to December 2020. The study inclusion criteria were: (1) Patients diagnosed with type B AD and underwent 3D printing-assisted stent graft fenestration; (2) Hospital stay > 7 days; (3) Age ≤ 90 years, life expectancy > 1 year (based on the hospital annual screening database); (4) had nutritional status (based on the parameters investigated in this study) within normal ranges prior to surgery. The exclusion criteria were: (1) Hospital stay > 2 months or underwent urgent procedures; (2) History of stroke or hemorrhage in organs in the past 3 months; (3) Complicated with immune system diseases, tumors, digestive system disease; (4) Severe heart, liver, kidney and other vital organ lesions. Based on these criteria, a total of 99 patients were eligible for this study. The patient screening process is shown in Fig. 1.

According to different number of fenestrations, the patients were divided into four groups: one fenestration (group A, n = 37), two fenestrations (group B, n = 16), three fenestrations (group C, n = 31) and four fenestrations (group D, n = 15). Additionally, using the criteria of 25 kcal/kg/day based on the actual body weight of the patients as the goal calories, the patients were divided into a Reached group and a Not reached group depending on whether their calories reached 80% of the goal calories via infusion on postoperative day 5 [10, 11]. This study was approved by the Ethics Committee of Nanjing Drum Tower Hospital, The Affiliated Hospital of Nanjing University Medical School (Approval Number: 2022-239-02) and informed consent was given by all patients.

### Collection of baseline data

The data collection form of this study was designed by the authors after consulting with relevant literature and experts. The form mainly included the general data of patients: age, sex, BMI, economic status, history of diabetes, history of smoking, and history of drinking.

### Collection of clinical variables

Clinical variables included operation duration, intraoperative blood loss, preoperative and postoperative NRS2002 score [12], as well as white blood cell (WBC) and C-reactive protein (CRP) levels on postoperative days 1 and 5.

### Statistical analysis

Statistical software SPSS 22.0 was used for data analysis. Measurement data that conformed to a normal distribution are presented as mean ± standard deviation (SD). The *t* test was performed for comparison between two groups, and one-way analysis of variance for comparison among multiple groups. Variables with skewed distribution are expressed as M (Q1, Q3), for which the Kruskal–Wallish test was used to compare multiple groups and the LSD test for pairwise comparison. Qualitative data are expressed as rate or constituent ratio, and means between groups were compared using the chi-square test. *P* < 0.05 was used to indicate a statistically significant difference.

## Results

### Baseline characteristics

Of the 99 patients who underwent 3D printing-assisted stent graft fenestration, there was no significant statistical difference in gender composition, mean age, BMI, underlying diseases (diabetes), economic status and living habits (smoking history, drinking history) between groups A, B, C, D (*P* > 0.05; Table 1).

**Table 1:** Baseline data of patients with aortic dissection in the four groups

Variable	Total (n = 99)	Group A (n = 37)	Group B (n = 16)	Group C (n = 31)	Group D (n = 15)	*P* value
Sex, n (%)						0.705
Male	80 (80.8)	31 (83.8)	14 (87.5)	24 (77.4)	11 (73.3)	
Female	19 (19.2)	6 (16.2)	2 (12.5)	7 (22.6)	4 (26.7)	
Age	59.7 ± 12.7	62.2 ± 14.3	62.0 ± 10.6	58.7 ± 11.5	53.5 ± 11.7	0.127
Body mass index (BMI)	24.2 ± 3.3	24.5 ± 3.5	25.5 ± 2.7	23.9 ± 3.1	22.8 ± 3.7	0.126
Economic status, n (%)						0.659
Medical insurance	75 (75.8)	27 (73.0)	14 (87.5)	22 (71.0)	12 (80.0)	
Self-pay	24 (24.2)	10 (27.0)	2 (12.5)	9 (29.0)	3 (20.0)	
Diabetes, n (%)						0.536
No	89 (89.9)	34 (91.9)	13 (81.2)	29 (93.5)	13 (86.7)	
Yes	10 (10.1)	3 (8.1)	3 (18.8)	2 (6.5)	2 (13.3)	
History of smoking, n (%)						0.812
No	74 (74.7)	26 (70.3)	12 (75.0)	25 (80.6)	11 (73.3)	
Yes	25 (25.3)	11 (29.7)	4 (25.0)	6 (19.4)	4 (26.7)	
History of drinking, n (%)						0.118
No	75 (75.8)	25 (67.6)	12 (75.0)	28 (90.3)	10 (66.7)	
Yes	24 (24.2)	12 (32.4)	4 (25.0)	3 (9.7)	5 (33.3)	

Measurement data are expressed as mean ± SD, while qualitative data are expressed as n (%). group A, one fenestration; group B, two fenestrations; group C, three fenestrations; group D, four fenestrations

### Analysis of clinical variables in patients with different numbers of fenestrations

As shown in Table 2, there were significant differences in operation duration and intraoperative blood loss among the four groups (*P* < 0.05). Specifically, operation duration in group A (2.76 ± 1.45) was significantly shorter than that of group B (4.36 ± 2.25), group C (5.36 ± 2.39) and group D (5.10 ± 1.53) (*P* < 0.05). The intraoperative blood loss of group A (50 [50, 150]) was significantly lower than that of groups C (100 [100, 300]) and D (500 [100, 500]), but no marked difference in intraoperative blood loss was found between groups A and B (*P* > 0.05), groups C and D (*P* > 0.05). No significant differences were identified among the four groups in preoperative and postoperative NRS scores (*P* > 0.05). In terms of nutrition status, patients in group A had a significantly higher rate of reaching the nutritional requirements (48.6%) compared with patients in groups B (18.8%), C (19.4%) and D (6.7%) (*P* = 0.009), but little difference was found among groups B, C, and D (*P* > 0.05).

**Table 2:** Relationship between different number of fenestrations and clinical variables of patients

Variables	Total (n = 99)	Group A (n = 37)	Group B (n = 16)	Group C (n = 31)	Group D (n = 15)	*P* value
Operation duration	4.24 ± 2.24	2.76 ± 1.45^bcd^	4.36 ± 2.25	5.36 ± 2.39	5.10 ± 1.53	< 0.001
Preoperative NRS score, n (%)						0.247
0	46 (46.5)	19 (51.4)	7 (43.8)	13 (41.9)	7 (46.7)	
1	30 (30.3)	13 (35.1)	3 (18.8)	9 (29.0)	5 (33.3)	
2	13 (13.1)	5 (13.5)	3 (18.8)	5 (16.1)	0 (0.0)	
3	5 ( 5.1)	0 (0.0)	2 (12.5)	1 (3.2)	2 (13.3)	
4	5 ( 5.1)	0 (0.0)	1 (6.2)	3 (9.7)	1 (6.7)	
Postoperative NRS score, n (%)						0.159
2	7 ( 7.1)	5 (13.5)	0 (0.0)	2 (6.5)	0 (0.0)	
3	42 (42.4)	18 (48.6)	9 (56.2)	10 (32.3)	5 (33.3)	
4	37 (37.4)	13 (35.1)	5 (31.2)	11 (35.5)	8 (53.3)	
5	6 ( 6.1)	1 (2.7)	1 (6.2)	4 (12.9)	0 (0.0)	
6	7 ( 7.1)	0 (0.0)	1 (6.2)	4 (12.9)	2 (13.3)	
Intraoperative blood loss (ml)	100 (50, 300)	50 (50, 150)^c,d^	300 (50, 525)	100 (100, 300)	500 (100, 500)	0.002
Nutritional status, n (%)						0.009
Met nutrition requirement	28 (28.3)	18 (48.6)^b,c,d^	3 (18.8)	6 (19.4)	1 (6.7)	
Failed to meet nutrition requirement	71 (71.7)	19 (51.4)	13 (81.2)	25 (80.6)	14 (93.3)	

Operation duration is expressed as mean ± SD, preoperative and postoperative NRS score as n (%), and intraoperative blood loss as M (Q1, Q3). ^bcd^ represents a significant difference between group A and the other three groups. group A, one fenestration; group B, two fenestrations; group C, three fenestrations; group D, four fenestrations

### Changes of laboratory parameters after operation in patients with calorie provided reaching or not reaching the nutrition requirement

Of the 99 patients assessed, 28 (28.3%) met the nutrition requirement on postoperative day 5 (Table 2). Additionally, WBC count and CRP concentration on postoperative day 5 in the Reached group were significantly lower than those on postoperative day 1 (*P* < 0.05), and the proportion of patients with abnormal WBC count was also significantly lower than that on postoperative day 1. CRP concentration on postoperative day 5 in the Not reached group was significantly lower than that on postoperative day 1 (*P* < 0.05), but WBC count was not markedly different from that on postoperative day 1 (*P* = 0.236). In addition, the Not-reached group showed no significant difference in the number of patients with abnormal WBC counts between postoperative days 1 and 5 (*P* = 0.253) (Table 3).

**Table 3:** Comparison of white blood cells and C-reactive protein on postoperative days 1 and 5

Variables	Time	Reached group (n = 29)	Not reached group (n = 70)	*P* value
WBC	D1	11.70 (8.30,16.50)	12.25 (8.20,16.15)	0.479
	D5	10.20 (7.20,13.80)	11.15 (8.78,16.15)	< 0.001
	*P*	< 0.001	0.236	
CRP	D1	83.90 (54.30,120.50)	110.20 (72.53,165.03)	< 0.001
	D5	63.70 (26.90,99.30)	75.50 (30.90,128.80)	< 0.001
	*P*	< 0.001	0.002	
Abnormal WBC count	D1	149 (59.60)	132 (76.70)	0.029
	D5	121 (48.40)	6123 (71.50)	< 0.001
	*P*	0.006	0.253	

WBC count is expressed as n (%), and CRP concentration as M (Q1, Q3). WBC, white blood cell; CPR, C-reactive protein; D1, day 1; D5, day 5

## Discussion

3D printing-assisted stent graft fenestration technique is a new attempt developed by clinical workers in recent years to solve the problem of insufficient landing zone. With this technique, the anatomical structure and positional relationship between branch arteries and aneurysm can be visually displayed [12]. The 3D printing method can also reconstruct the affected visceral arteries, ensure the blood supply of target organs, and significantly reduce potential damage to target organs and patient mortality [13–15]. In terms of nutritional status, we found the rate of reaching the nutrition requirement was higher in patients with one fenestration (48.6%), while the rate among the other three groups was not significantly different. Patients with single fenestration accounted for 64.29% of all patients reaching nutrition requirements. Our findings further demonstrated that multiple fenestrations were associated with a low rate of reaching nutrition requirements. Therefore, more attention should be paid to the postoperative nutritional status of patients with multiple fenestrations, and a refined nutritional program is recommended as early as possible to promote the postoperative recovery of these patients. In terms of surgery-related parameters, group A (one fenestration) had the shortest operation duration and the least intraoperative blood loss. Group D (four fenestrations) had the most intraoperative blood loss. However, there was no significant statistical difference in the intraoperative blood loss between groups B, C and D, and the number of fenestrations did not increase positively with the intraoperative blood loss.

Patients undergoing surgery for AD have increased postoperative infection rates due to surgery trauma and decreased autoimmune function [16]. Long-term general anesthesia and gastrointestinal dysfunction can lead to the secretion of a large number of inflammatory factors, triggering systemic inflammatory response syndrome, which is an important cause of multiple organ function damage [17]. Inflammation can disrupt the metabolism of the patients, causing an increase in insulin resistance and reduction in appetite [18, 19], thus, inhibiting intestinal smooth muscle contraction and absorption of nutrients into cells and altering their gastrointestinal functions. WBC and CRP are direct reflections of inflammation and infection, and CRP is also a marker of acute inflammation. Gariballa et al. found that energy intake was significantly lower in patients with higher CRP concentrations [20]. In our study, the WBC count, CRP concentration and the number of patients with abnormal WBC count on postoperative day 5 in the Reached group were significantly lower than those on postoperative day 1, while WBC count and the number of patients with abnormal WBC count in the Not reached group on postoperative day 5 were not significantly different from those on postoperative day 1. These results suggest that in the first 5 days after surgery, the inflammatory response was alleviated in the Reached group but not in the Not reached group. Persistent high inflammatory levels in patients in the Not reached group may have a negative impact on gastrointestinal motility and function, affecting the digestion and absorption of nutrients and exacerbating nutritional intolerance. In summary, failure to reach the nutrition requirement may be related to the inflammatory status, especially persistent and serious inflammatory response, so it is speculated that the inflammatory status is an important factor affecting the nutrition status of patients.

Malnutrition after surgery for AD slows the rate of wound healing, increases the incidence of infection, prolongs hospital stay, increases medical costs, and impedes patient recovery [21]. Early recovery of immune function can reduce postoperative complications, which is of great significance for improving the prognosis of patients undergoing surgery for AD and is key to promoting patient rehabilitation [22, 23]. Additionally, timely and effective nutritional support for patients after aortic fenestration can improve their immune functions and reduce inflammatory stress responses, thus boosting the rate of reaching the nutrition requirement, reducing postoperative complications, and consequently improving treatment outcomes and patient recovery and quality of life.

The study limitations included the unavailability of data, such as dynamic changes in inflammatory factor levels and continuous gastrointestinal function scores, which forced us to select only WBC and CRP for analysis. Further, the single-center retrospective nature of this study and the limited number of patients analyzed, especially for group assessment, which might have led to a certain level of bias; therefore, large cohort and more in-depth studies using prospective settings are still required to confirm the significance of the 3D technique on patients recovery.

## Conclusion

In summary, among patients with AD treated with 3D printing-assisted stent graft fenestration, those with multiple fenestrations have a low rate of reaching nutrition requirements (80% of goal calories), and in which inflammation might be related to nutritional status. Therefore, we suggest that nutrition status be evaluated and graded for patients with multiple fenestrations after the surgery. Additionally, diversified nutrition regimens should be implemented early to promote the patients’ postoperative recovery.

## Data Availability

The data that support the findings of this study are available from the corresponding author upon reasonable request.

## Abbreviations

AD: Aortic dissection
WBC: White blood cell
CRP: C-reactive protein
